# The psychological impact, risk factors and coping strategies to COVID-19 pandemic on healthcare workers in the sub-Saharan Africa: a narrative review of existing literature

**DOI:** 10.1186/s40359-022-00998-z

**Published:** 2022-12-01

**Authors:** Freddy Wathum Drinkwater Oyat, Johnson Nyeko Oloya, Pamela Atim, Eric Nzirakaindi Ikoona, Judith Aloyo, David Lagoro Kitara

**Affiliations:** 1 Uganda Medical Association (UMA), UMA-Acholi Branch, Gulu City, Uganda; 2 Moroto Regional Referral Hospital, Moroto, Uganda; 3 St. Joseph’s Hospital, Kitgum District, Uganda; 4 ICAP at Columbia University, Freetown, Sierra Leone; 5 Rhites-N, Acholi, Gulu City, Uganda; 6 Faculty of Medicine, Department of Surgery, Gulu University, P.O. Box 166, Gulu City, Uganda; 7 Harvard University, Cambridge, USA

**Keywords:** COVID-19 pandemic, Social support, Occupational health and safety, Mental health surveillance, Workplace organization

## Abstract

**Background:**

The ongoing COVID-19 pandemic has significantly impacted the physical and mental health of the general population worldwide, with healthcare workers at particular risk. The pandemic's effect on healthcare workers' mental well-being has been characterized by depression, anxiety, work-related stress, sleep disturbances, and post-traumatic stress disorder. Hence, protecting the mental well-being of healthcare workers (HCWs) is a considerable priority. This review aimed to determine risk factors for adverse mental health outcomes and protective or coping measures to mitigate the harmful effects of the COVID-19 crisis among HCWs in sub-Saharan Africa.

**Methods:**

We performed a literature search using PubMed, Google Scholar, Cochrane Library, and Embase for relevant materials. We obtained all articles published between March 2020 and April 2022 relevant to the subject of review and met pre-defined eligibility criteria. We selected 23 articles for initial screening and included 12 in the final review.

**Result:**

A total of 5,323 participants in twelve studies, predominantly from Ethiopia (eight studies), one from Uganda, Cameroon, Mali, and Togo, fulfilled the eligibility criteria. Investigators found 16.3–71.9% of HCWs with depressive symptoms, 21.9–73.5% with anxiety symptoms, 15.5–63.7% experienced work-related stress symptoms, 12.4–77% experienced sleep disturbances, and 51.6–56.8% reported PTSD symptoms. Healthcare workers, working in emergency, intensive care units, pharmacies, and laboratories were at higher risk of adverse mental health impacts. HCWs had deep fear, anxious and stressed with the high transmission rate of the virus, high death rates, and lived in fear of infecting themselves and families. Other sources of fear and work-related stress were the lack of PPEs, availability of treatment and vaccines to protect themselves against the virus. HCWs faced stigma, abuse, financial problems, and lack of support from employers and communities.

**Conclusion:**

The prevalence of depression, anxiety, insomnia, and PTSD in HCWs in sub-Saharan Africa during the COVID-19 pandemic has been high. Several organizational, community, and work-related challenges and interventions were identified, including improvement of workplace infrastructures, adoption of correct and shared infection control measures, provision of PPEs, social support, and implementation of resilience training programs. Setting up permanent multidisciplinary mental health teams at regional and national levels to deal with mental health and providing psychological support to HCWs, supported with long-term surveillance, are recommended.

## Introduction

When coronavirus disease 2019 (COVID-19) was declared a pandemic in March 2020, healthcare workers (HCWs) globally and in sub-Saharan Africa (SSA) were unprepared for the scale of the physical and mental health devastation that was to follow [1]. The impact of the COVID-19 pandemic on healthcare workers has been profound, characterized by death, disability, and untenable burden on mental health and well-being [2]. Factors impacting their mental health include high risks of exposure and infection, financial insecurity, separation from loved ones, stigma, difficult triage decisions, stressful work environment, scarcity of supplies including personal protective equipment (PPEs), exhaustion, traumatic experiences due to regular witnessing of deaths among patients and colleagues [2, 3]. Greenberg et al. [4] observed that the COVID-19 pandemic put healthcare professionals worldwide in an unprecedented situation, making difficult decisions to provide care for many severely ill patients with constrained or inadequate resources.

In almost all WHO regions, data indicates that infection rates among healthcare workers are higher than in the general population [5]. Scholars suggest that the end of the COVID-19 pandemic is not yet in sight. Neither are they sure about the virulence of the following variant when it appears as caseloads are still rising, with more than 621 million infections and 6.5 million deaths reported worldwide by 19th October 2022 [6]; mainly driven by the newer omicron variants. However, recently in October 2022, we received with gratitude a reassuring message from US President Biden declaring the end of the COVID-19 pandemic in the United States of America.

Meanwhile, previous studies found high levels of depression, anxiety, and PTSD in survivors among the general population and healthcare workers (HCWs) one-to-three years after the control of the SARS epidemic [7] and the 2014–2016 Ebola epidemic in West Africa [8]. In addition, recent surveys [9–14], reviews, and meta-analyses [15–18] are pointing to early evidence that a considerable proportion of healthcare workers have experienced stress, anxiety, depression, and sleep disturbances during the COVID-19 pandemic, raising concerns about risks to their long-term mental health.

Studies from the global north countries [19, 20], UK [21], USA [22], and in India [23], and China [24, 25] have shed light on the vulnerability that characterizes frontline healthcare workers during this pandemic, especially regarding their mental health and well-being. However, evidence in sub-Saharan Africa is scanty, and the pattern and prevalence of psychological disorders are not well understood.

Evidence from a systematic review by Pappa S et al. on 33,062 Chinese HCWs in April 2020 found a pooled prevalence rate of mental health problems among respondents; anxiety 23.2%, depression 22.8%, and insomnia 38.9% [26]. Similarly, Singapore study, Tan et al*.* [27], Li et al*.* [28], BMA [29] and in China [31] found high levels of psychological disorders among health workers.

Since the beginning of the pandemic, we found one systematic review involving 919 frontline HCWs, 3928 general HCWs, and 2979 medical students conducted in Africa from December 2019 to April 2020 [31]. The study by Chen J et al*.* reported a high prevalence of depression, anxiety, and insomnia among frontline HCWs in sub-Saharan Africa (SSA) at 45%, 51%, and 28%, respectively. In comparison, the prevalence of depression, anxiety, and insomnia among the general population was much lower at 30%, 31%, and 24%, respectively [31]. Furthermore, we found that only a few studies investigated protective and coping measures, given the many uncertainties surrounding the evolution of the COVID-19 pandemic [32]. Adequate data are needed to equip frontline HCWs and healthcare managers in sub-Saharan Africa to mitigate the medium and long-term adverse effects of the COVID-19 pandemic [33].

This review aimed to answer three questions (1) What is the psychological impact of the COVID-19 pandemic on HCWs in Sub-Saharan Africa?

(2) What are the associated risk factors during the COVID-19 pandemic?

(3) What interventions (mitigating and coping strategies) protect and support the mental health and well-being of HCWs during the ongoing crises and after the pandemic?

## Methodology

### Search methodology and article selection

This current article is a mixed-method narrative review of existing literature on mental health disorders, risk factors, and interventions relevant to the COVID-19 pandemic on HCWs in sub-Saharan. A search on the PubMed electronic database was undertaken using the search terms "novel coronavirus", "COVID-19", "nCoV", "mental health", "psychiatry", "psychology", "anxiety", "depression" and "stress" in various permutations and combinations.

### Search processes

We conducted a comprehensive literature search on original articles published from March 2020 to 30 April 2022 in electronic databases of Embase, PubMed, Google Scholar, and the daily updated WHO COVID-19 database. Our search terms included but were not limited to ('COVID-19'/exp OR COVID-19 OR 'coronavirus'/exp OR coronavirus) AND ('psychological'/exp OR psychological OR 'mental'/exp OR mental OR 'stress'/exp OR stress OR 'anxiety' OR anxiety OR 'depression' OR depression OR 'post-traumatic' OR 'post-traumatic'/exp OR 'trauma' OR 'trauma'/exp) OR Health care workers, medical workers of health care professionals, sub-Saharan Africa, for Embase. ("COVID-19" [All Fields] OR "coronavirus" [All Fields]) AND ("Stress, Psychological" [Mesh] OR "mental" OR "anxiety" OR "depression" OR "stress" OR "post-traumatic" OR "trauma") for PubMed, for the WHO COVID-19 database, and ("COVID-19" OR "coronavirus") AND ("Psychological" OR "mental" OR "anxiety" OR "depression" OR "stress" OR "post-traumatic" OR "trauma") for Google Scholar. On reviewing the above citations, twelve articles met the inclusion criteria relevant for this review and are in Table 1. All twelve articles were cross-sectional, with one qualitative and the others quantitative observational studies.

**Table 1:** Study characteristics and outcome measures

s/n	Authors	Country	Study design	Sampling Procedure/Sample Size (n)	Instrument Applied	Main outcome measures
1	Muzyamba et al*.* [35]	Uganda	Qualitative study Cross-sectional Online self-administered questionnaires	Random selection n = 50 Frontline HCWs, 56% males	Qualitative Online survey tools	Depression, anxiety, and PTSD Risks: long working hours, lack of equipment, PPEs, Testing kits, lack of sleep, exhaustion, high death rates, death of colleagues, high rates of transmission Coping strategies: Family networks, community networks, helps from family, responsibility to society, assistance from community members, availability of assistance from strangers and symbiotic nature of assistance in community
2	Sagaon Teyssier et al*.* [39]	Mali	Quantitative study Cross-sectional Non-frontline HCWs, involved in HIV care	Non-probability sampling n = 135(60.7% male, mean age 40yrs)	PHQ-9, (20–27); GAD-7, (0–21), 7 ISI (0–28)	Prevalence: Depression at 71.9%, anxiety at 73.5%, and Insomnia at 77% Risk factors: female, lack of PPEs, and lack of nurses 21.5% had severe depression. Depression was 60% more likely in females than males
3	GebreYesus FA et al*.* [36]	Ethiopia	Quantitative study Cross-sectional, Self-administered questionnaires	Probability sampling. Public hospital n = 322, Response rate 96.5% Males 51.9%	PHQ-9, PSS-10	Prevalence: Depression at 25.8%, anxiety at 36%, Stress at 31.4%. overall prevalence of MHD was 36% Risk factors: age, gender, education, low income, presence of infected member of a family, living with family, and occupation
4	Mulatu HA, et al. [37]	Ethiopia	Quantitative study Cross-sectional, self-administered questionnaires	Probability sampling. St. Paul 2^nd^ Largest Public Hospital Tertiary hospital. Addis Ababa n = 420 response rate 97. Males 58.6% Frontlines HCPs 70.5%	PHQ-9, GAD-7, ISI, IES-R	Prevalence: Depression at 20.2%, anxiety at 21.9%, insomnia at 12.4%, and distress at 15.5% Risk factors: Married, frontline workers, exposure to COVID-19 cases, stigma, infected family members, work shift arrangement with longer hours, lack of logistic supports, PPEs & poor or lack of accommodation at the workplace
5	Ayalew, et al. [38]	Ethiopia	Quantitative study Cross-sectional study design. Self-administered questionnaires	Probability sampling, Frontline & Non-frontline HCWs in four Public Hospitals, n = 387, response rate 91.7% Males 58.7%, Southern Ethiopia	IES-R	Prevalence: PTSD at 56.8% Significant risk factors were age, working environment, professions, female, married, and nurse. In patients, workers, emergency workers, and ICU, Independent predictors were females, married and nurses
6	Jemal K, et al*. *[40]	Ethiopia	Quantitative study Cross-sectional Self-administered Questionnaires	Probability sampling. Facility based study n = 417, Response rate 98.6%. North Shoa Zone, Oromiya	PHQ-9, GAD-7, ISI, IES-R	Prevalence: Depression at 16.3%, anxiety at 30.7%, Insomnia at 15.9%, and Stress at 58%
7	Chekole YA., et al. [41]	Ethiopia	Quantitative study Cross-sectional Self-administered questionnaires	Probability sampling, Institutional HCPs n = 244 response rate 100%, Males 66%	PSS-10	PTSD Prevalence was 51.6% Risk factors were age, and educational qualifications Age and profession were independent predictors of stress. Frontline HCWs had a strong statistical association with stress, nurses, pharmacists, frontlines, and master’s degree
8	Asnakew et al*. *[43]	Ethiopia	Quantitative study Cross-sectional study design, Multicentre self-administered questionnaires to Hospital workers	Probability sampling, n = 396 response rate 93.6%. Males 69.2% females30.8% Northwest Ethiopia	IES-R-22	PTSD prevalence was 55.1% Risk factors were lack of standard PPEs, age > 40, medical illness, females, perceive stigma, history of mental illness, poor social support, and being a physician
9	Asnakew et al*.* [42]	Ethiopia	Quantitative study Cross-sectional study design Self-administered questionnaires Multicentre in eight hospitals	Probability sampling. n = 419; response rate 99.1%; Males 69%, females 31% Nurses 52% (218). Public Hospitals South Gondar Northwest Ethiopia	DASS-21 Oslo 3 items (OSS-3 for social support)	Prevalence: Depression at 58.2%, anxiety at 64.7%, stress at 63.7% Risk factors: Frontliners, Chronic medical illness, mental illness, contact with COVID-19 case, poor social support, and females
10	Ayalew et al*. *[45]	Ethiopia	Quantitative study Cross-sectional study design Self-administered questionnaires Multicentre	Probability sampling. n = 387, public hospitals. Frontlines & Non-frontlines Males 58.7% Southern Ethiopia	DASS-21	Prevalence: Depression at 50.1%, anxiety at 55%, stress at 38.5% Risk factors: Anxiety; frontliners, in patient, HCWs, ICU, nurses, emergency workers, OPD, Laboratory technician, pharmacists, older age, females, and the married Depression risks: female, married, living alone, nurses, and inpatient workers, Stress risks: females, nurses, inpatients, living alone, and the married
11	Kounou KB et al. [44]	Togo	Quantitative study Cross sectional study design. Hospital-based Self-administered questionnaires	Non-probability n = 62, females 56.5% Lome, Togo	PHQ-9, GAD-7, PMS-9	Prevalence: Anxiety at 62.9%, and depression at 51.6% Risk factors: profession, workstation, women, and medical illness
12	Keubo et al*.* [47]	Cameroon	Quantitative study Cross-sectional study design. Self-administered questionnaires Hospital based data NGO Institutions	Non-probability n = 292, females 54.5%, Cameroon	HADS	Prevalence: Depression at 43.5% and anxiety at 42.2% The fear of infection and death were associated with depression & anxiety. Assistant nurses had the highest prevalence of depression & anxiety

Measures’ descriptions: Depression: *PHQ-9* Patient Health Questionnaire. Anxiety: *GAD-7* Generalised Anxiety Disorder Questionnaire, Depression & Anxiety: *DASS-21* Depression, Anxiety and Stress Scale, Sleep: *ISI* Insomnia Severity Index; *IES-R* Impact of Event Scale; *HADS* Hospital Anxiety and Depression scale, *PSS-10* Perceived Stress Scale; *PMS-9* Premenstrual Syndrome Scale; *OSS* Oslo 3 items for social support

### Eligibility criteria

We included original qualitative and quantitative studies examining the risk factors, psychological impact of COVID-19 and coping strategies of healthcare workers (HCWs) in sub-Saharan Africa during the COVID-19 pandemic. We excluded studies if they were.

1. Not reported in the English language 2. Studies which were not primary research 3. Studies that had not been published in a peer-reviewed journal 4. Studies that did not include data on HCWs’ mental health or psychological well-being 5. Duplicate studies 6. not using validated instruments to measure the risks and psychological impact.

FWDO performed the search of articles. DLK reviewed the articles involving screening of titles, followed by examination of abstracts. The potential articles identified were further reviewed in full text to examine their eligibility. In addition, four of the authors independently reviewed the full articles to abstract the relevant data required for the review. Thereafter, a meeting to harmonise findings were done and presented in a report.

### Data extraction and appraisal of the study

We extracted information from each study, including author, study population, year of publication, country, socio-demographic characteristics, sample size, response rate, gender proportion, age, and study time, areas assessed, the validated instrument used and the prevalence. The appraisal involved assessing the research design, recruitment of respondents, inclusion and exclusion criteria, reliability of outcome determination, statistical analyses, ethical compliance, strengths, limitations, and clinical implications of the articles.

Our review protocol was not registered on PROSPERO because of the significant variation in the methodologies of the articles used in the review. The results precluded using a meta-analytic approach and made a narrative review the most suitable for this work. In addition, we did not use the Cochrane Collaboration GRADE method to assess the quality of evidence of outcomes included in this narrative review. Instead, we used the Strengthening the Reporting of Observational studies in Epidemiology (STROBE) 22 items checklist to gauge the quality of the twelve articles included in this review. We qualitatively validated the articles based on additional considerations namely study design, sample sizes, sampling procedures, response rates, statistical methods used, measures taken by the authors to deal with bias and confounding factors and ethical consideration.

### Definition of healthcare worker (HCW)

For this narrative review, we adhered to the Centres for Disease Control and Prevention (CDC) definition of HCWs, which includes physicians, nurses, emergency medical personnel, dental professionals and students, medical and nursing students, laboratory technicians, pharmacists, hospital volunteers, and administrative staff [34].

## Results

### Search results

The search found twenty-three studies of interest. Full texts of potentially relevant studies underwent eligibility assessment, and twelve articles met the inclusion criteria for this narrative review.

### Study characteristics

The twelve articles comprised eleven quantitative and one qualitative study. The common mental health conditions assessed were depression, anxiety, perceived stress, and post-traumatic stress disorder (PTSD). The coping strategy, perceived health status, health distress (including burnout), insomnia, and perceived stigma were also assessed [35, 36]. The total number of respondents in these studies was 5,323. The qualitative study had fifty respondents [35], while the most significant number of participants, 420 was recorded in one of the quantitative studies from Ethiopia [37]. The questionnaire response rates varied between 90%-100%, with most studies dominated by male respondents at 51.9%-69.2% [38]. Nurses were the commonest study population, followed by doctors, pharmacists, and laboratory technicians, and no study involved non-HCWs of facilities. Most papers utilized probability sampling procedures, and four quantitative studies used non-random sampling procedures limiting generalizability of their findings and increasing the risk of selection bias. Eight studies were from Ethiopia, and one was from Cameroon, Uganda, Mali, and Togo, respectively (Table 1). Most studies were conducted in urban tertiary public hospitals, university teaching hospitals, and rural and urban general hospitals, including primary care facilities operated by Non-Governmental Organizations (NGOs) for example in Mali [39]. Several validated tools assessed depression, anxiety, insomnia, stress, and PTSD (Table 1).

Table 1 provides an overview of the studies selected and validated instruments used to measure psychological disorders.

Table 2 provides comparisons with studies conducted outside of sub-Saharan Africa.

**Table 2:** Comparisons of the prevalence of mental health disorders among HCWs in different regions

s/no	Authors/Country/Regions	Study design	Population	Period	Outcome
1	Chen J et al. Africa	Systematic Review/Meta-analysis	Frontline/General/ HCWs/4,847	Dec.2019–April 2020	Anxiety 51%, depression 45%, and insomnia 28%
2	Pappa S et al. China	Systematic Review/Meta-analysis	HCWs, 33,062	17 April 2020	Anxiety 23.2%, depression 22.8%, and Insomnia 38.9%
3	Li Y, et al. China	Systematic Review/Meta analysis	HCWs 33,062	17 April 2020	Anxiety 22.1%, depression 21.7%, and PTSD 21.5%
4	Basreeqa SB et al. China	Systematic Review/Meta analysis	General Population Frontline/General HCWs. 62,382	First six months of 2020	Anxiety 48.1%, depression 26.9%, and Stress 48.1%
5	Preti E et al., Asia, Middle East, Europe, USA	Rapid Review	HCWs	March 2020	Anxiety 45%, depression 27.5–50.7%, Stress 18.1–80.1%, Insomnia 34–36%, and PTSD 11–73.4%
6	Lai J. China	Cross-sectional	HCWs	First six months of 2020	Anxiety 44.6%, depression 50.4%, distress 71.5%, and insomnia 34%
7	Tan BYQ. Singapore	Cross-sectional	General population /HCWs	First six months of 2020	Anxiety 14.5%, depression8.9%, Stress 6.6%, and PTSD 7.7%
8	Consolo U et al. Italy	Cross-sectional	HCWs	First six months of 2020	Anxiety 46.4%, depression 70.2%, and stress 42.4%
9	Gilleen J et al. UK	Cross-sectional	HCWS	First six months of 2020	Anxiety 33%, Depression 28%, and PTSD 15%
10	Shacther A, et al. NY, USA	Cross-sectional	HCWs	First six months of 2020	Anxiety 33%, depression 48%, and stress 57%
11	Urooj U, et al. Pakistan	Cross-sectional	HCWs	First six months of 2020	Anxiety 86%, depression 58%, and stress 28.8%
12	Wilson W, et al. India	Cross-sectional	HCWs	First six months of 2020	Anxiety 17.7%, depression 11.6%, and stress 3.7%
13	Elhadi M, et al. Libya	Cross-sectional	HCWs	Early 2022	Anxiety 46.7%, and depression 56.3%

*HCWs* Healthcare workers; *PTSD* posttraumatic stress disorder

Table 3 provides information on studies showing the classification of psychological outcomes.

**Table 3:** Classification of studies according to psychological outcomes

Psychological Outcome	Studies	Measurement tools	Prevalence
Anxiety	[34–36, 38, 39, 42–45]	GAD-7, DASS-21, HADS	21.9–73.5%
Depression	[34–36, 38, 39, 42–45]	PHQ-9, GAD-7, HADS	16.3–71.9%
PTSD	[34, 37, 40, 41]	IES-R, PSS-10	51.6–56.8%
Stress	[35, 36, 42–44]	IES-R, OSS, PSS-10	15.5–63.7%
Insomnia	[35, 36, 38, 39]	ISI	12.4–77%
General psychological disorders	[35]	PSS, PHQ-9	36%

Measures’ descriptions: Depression: *PHQ-9* Patient Health Questionnaire. Anxiety: *GAD-7* Generalised Anxiety Disorder Questionnaire, Depression & Anxiety: *DASS-21* Depression, Anxiety and Stress Scale, Sleep: *ISI* Insomnia Severity Index; *IES-R* Impact of Event Scale; *HADS* Hospital Anxiety and Depression scale, *PSS-10* Perceived Stress Scale; *PMS-9* Premenstrual Syndrome Scale; *OSS* Oslo 3 items for social support

Table 4 are studies showing risk factors associated with psychological disorders.

**Table 4:** Studies showing risk factors associated with psychological disorders

Variables	PTSD	Anxiety	Depression	Insomnia	Stress	General psychological disorders
Age	[38, 41, 42] *	[36] *	N/A	N/A	[36] *	[36] *
Female Gender	[38, 42]*	[36, 39, 43–45] *	[36, 39, 43–45] *	[37, 39] *	[36, 43] *	N/A
Marital status	[38] *	[37] *	[37] *	[37] *	N/A	N/A
Education	[41] *	N/A	N/A	N/A	N/A	N/A
Income	N/A	[36] *	[36] *	[36] *	[36] *	N/A
Physicians	N/A	[41, 42] *	[37, 43, 44] *	[37, 43–45] *	[36, 37] *	N/A
Nurses	[38, 41] *	[35, 37, 44, 45] *	[35, 37, 44, 45] *	[36, 37] *	[35–37] *	N/A
Pharmacists	[38, 41] *	[36, 37, 39, 44, 45] *	[36, 37, 39, 44, 45] *	[36, 37] *	[36, 37] *	N/A
Lab. Tech	[38, 41] *	[36, 37, 39, 44, 45] *	[36, 37, 39, 44, 45] *	[36, 37] *	[35–37, 45] *	N/A
Frontline workers	[38, 41, 42] *	[35–39, 41–45] *	[35–39, 41–45] *	[36, 37] *	[35–37, 45] *	N/A
Stigma	[42] *	[37] *	[37] *	[37] *	[37] *	[37] *
Medical illness	[42] *	[37, 43, 44] **	[37, 43, 44] *	[37] *	[37, 43] *	N/A
Mental Illness	[42] *	[43, 44] *	[43, 44] *	N/A	[43] *	N/A
Lack of PPEs	[38, 41, 42] *	[35–39, 41–45] *	[35–39, 41–45] *	[35–37] *	[35–37] *	N/A
Contact with COVID-19 cases	[38, 41, 42] *	[35–37] *	[35–37] *	[35–37] *	[35–37, 43] *	N/A
Poor Social support	[42] *	[37, 43] *	[37, 43] *	[37, 43] *	[43] *	N/A
Living Alone	N/A	[45] *	[45] *	[45] *	N/A	[45]
Living with a family	N/A	[36] *	[36] *	N/A	[36] *	N/A
Infected family member	N/A	[36, 37] *	[36, 37] *	[36, 37] *	[36, 37] *	N/A

*positive association; N/A No association

Table 5 are studies that identified protective factors for psychological disorders.

**Table 5:** Studies that identify protective factors for psychological disorders

Variables	PTSD	Anxiety	Depression	Insomnia	Stress
Availability of PPEs	[35, 38, 41, 42]	[35–37, 42, 43]	[35–37, 42, 43]	[35–37, 42, 43]	[35–37, 42, 43]
Experience	[35, 42]	[37, 42, 45]	[37, 42, 45]	[37, 42, 45]	[37, 42, 45]
Training/orientation	[35, 42]	[37, 42, 45]	[37, 42, 45]	[37, 42, 45]	[37, 42, 45]
Safety of Family	[35]	[35, 36]	[35, 36]	[35, 36]	[35, 36]
Availability of testing kits	[35]	[35]	[35]	[35]	[35]
Work shifts arrangement	[35]	[37]	[37]	[37]	[37]
organizational support	[35]	[37]	[37]	[37]	[37]
Online Psychological support	[35, 42]	[37, 42]	[37, 42]	[37, 42]	[37, 42]
Better income	[35]	[36]	[36]	[36]	
Strong Social Support	[35, 42]	[35–37, 42, 43]	[35–37, 42, 43]	[35–37, 42, 43]	[35–37, 42, 43]
Community Support	[35, 42]	[35, 43]	[35, 43]	[35, 43]	[35, 43]

*PTSD* posttraumatic stress disorder

### Risks of bias and confounding factors

Most articles selected were cross-sectional studies that employed probability sampling procedures (Table 1). Cross-sectional study design minimized selection biases, but many used structured questionnaires, including online self-administered questionnaires, which increased bias due to social desirability. It was not clear how confounding variables were controlled in five papers reviewed [38–40, 43, 45] leading to excessive and perhaps inappropriate determination of associations.

### Socio-demographic factors

#### Age

In this review, the mean age of the respondents ranged between 23 and 35 years, and predominantly males. Age was associated with anxiety, and stress symptoms in 6(50%) of all the studies reviewed [35, 37, 40–42, 44]. An age of over 40 years was associated with moderate to severe symptoms of PTSD. Two studies concluded that respondents aged over 40 years were more likely to develop PTSD symptoms than their younger counterparts [37, 41].

#### Gender

Female gender was significantly associated with depression, anxiety, and stress symptoms among HCWs in seven studies reviewed [36–38, 41–43]. Many studies found that being female, married, and a nurse were independent predictors of stress symptoms. Moreover, sex, age, marital status, type of profession, and working environment were significant factors for PTSD symptoms [37, 41]. However, one study in Ethiopia found that the odds of depression were twice higher among male healthcare providers than among female healthcare providers [35].

### Psychological impact on healthcare workers

Most studies reviewed directly assessed the prevalence of depression, anxiety, stress, insomnia, and PTSD in HCWs. Common causes of anxiety, fear, or psychological distress that health professionals reported were: lack of access to PPEs and other equipment, being exposed to COVID-19 at work and taking the infection home to their families, uncertainties that their organization will support/take care of their personal and family needs if they got infection, long working hours, death of colleagues, lack of social support, stigmatization, high rates of transmission and poor income [35–45]. However, the prevalence of mental health symptoms exhibited great variations for example depressive symptoms were examined in nine studies [35–37, 39, 43–46], and varied between 16.3% and 71.9% among HCWs [38, 39].

In addition, nine other studies reported high prevalence of anxiety symptoms among HCWs [35–37, 40, 43–47] which varied between 21.9% and 73.5% [36, 39]. Five studies investigated HCWs' perceived stress during the pandemic; 15.5%-63.7% of HCWs reported high levels of work-related stress [35–37, 43, 45]. Three studies reported 12.4–77% of HCWs experienced sleep disturbances during the COVID-19 pandemic [37, 39, 40].

Post-traumatic stress disorder (PTSD) was in three studies [38, 41, 42], and the prevalence of PTSD-like symptoms varied between 51.6 and 56.8% in HCWs [38, 41]. A qualitative study from Uganda reported high symptoms of depression, anxiety, and PTSD among HCWs [35]. Additionally, factors that increased the risk of PTSD symptoms were for example, working in emergency units and being frontline workers. Furthermore, many studies found that frontline HCWs had increased symptoms of mental disorders and being a frontline worker was an independent risk factor for depression, anxiety, and PTSD [36–46].

### Risk factors associated with adverse mental health outcomes

The qualitative study from Uganda reported the factors associated with mental disorder symptoms among HCWs. These were long working hours, lack of equipment (PPEs, testing kits), lack of sleep, exhaustion, high death rates, death of colleagues, and a high COVID-19 transmission rate among HCWs [35]. Lack of equipment (PPEs, ventilators, and testing kits), overworking, and lack of logistic support were in Ethiopian studies [36–42, 45]. Most studies identified several risk factors for adverse mental health outcomes among respondents for example those with medical and mental illnesses, contacts with confirmed COVID-19 patients, and poor social support which were significantly associated with depression [42, 43]. Other factors were females, nurses, married, frontline workers, ICU, emergency units, living alone, and lack of social support [35, 37–45]. Too, participants’ families with chronic illnesses, had contacts with confirmed COVID-19 cases, and poor social support were significantly associated with anxiety. Other risk factors associated with anxiety include exhaustion, long working hours, frontline workers, emergencies, nurses, pharmacists, laboratory technicians, married, older, younger, living alone, being female, working at general and referral hospitals, and perceived stigma. In addition, participants’ families with chronic illnesses, those who had contacts with confirmed COVID-19 cases, and those with poor social support were predictors of stress during the COVID-19 pandemic [37, 38, 40–43, 45]. Other stress symptoms include having a medical illness, a mental illness, being a frontline worker, married, nurse, female, pharmacist, laboratory technician, physician, older age, lack of standardized PPE supply, low incomes, and living with a family [36, 37, 40–45]. Healthcare providers with low monthly incomes were significantly more likely to develop stress than those with high monthly incomes [38]. In addition, participants living alone, living with a family, and being married were associated with symptoms of psychological disorders among HCWs [36–38, 45]. Overall, the risk factors for adverse psychological impacts are categorized in three thematic areas (i) occupational, (ii) psychosocial, and (iii) environmental aspects.

### Occupational factors

Most studies showed that frontline HCWs, nurses, doctors, pharmacists, and laboratory technicians had significantly higher levels of mental health risks compared to non-frontline HCWs [35–38, 40, 42, 43, 45]. They experienced higher frequency of insomnia, anxiety, depression, and somatization than non-frontline medical HCWs. In contrast, Mali [39] and Cameroon [46] studies found a higher prevalence of depression, anxiety, and PTSD in non-frontline HCWs [39, 46]. However, among HCWs, physicians were 20% less likely to develop mental health disorders than nurses, pharmacists, and laboratory technicians [39]. In addition, healthcare workers with low monthly incomes had higher symptoms of depression, anxiety, stress, and insomnia [37].

#### Healthcare groups

Five studies found that being a nurse was associated with worse mental disorders than doctors [36, 37, 40, 44, 45].

#### Frontline staff with direct contact with COVID-19

Most papers in the review found that being in a “frontline” position or having direct contact with COVID-19 patients was associated with higher level of psychological distress [35–38, 40, 42, 43, 45]. In addition, studies found that contact with COVID-19 patients was independently associated with an increased risk of sleep disturbances [40, 46]. Moreover, HCWs who had contact with confirmed COVID-19 cases were more likely to develop depression, anxiety, and stress symptoms than those who had no contact with COVID-19 patients [36–38, 43, 45].

#### Lack of personal protective equipment (PPEs)

Most studies reported that the lack of PPEs was associated with higher symptoms of depression, anxiety, stress, and insomnia, while its availability was associated with fewer mental disorder symptoms [35–46]. In Mali, workers from centres that provided facemasks were 51% less likely to suffer from depression, 62% less likely to develop anxiety, and 45% less likely to develop insomnia [39]. In Ethiopia, the odds of developing post-traumatic stress disorder were much higher among HCWs who did not receive standardized PPEs supplies than those who had [38, 41, 42]. In Uganda, the lack of PPEs was associated with depression, anxiety, and PTSD [35].

#### Heavy workload

Longer working hours, increased work intensity, increased patient load, and exhaustion were risk factors in Ugandan [35] and Ethiopian studies [36].

### Psychosocial factors: perceived stigma and fear of infection

The fear of infection was in the qualitative study from Uganda [35], one quantitative study from Cameroon [47] and seven cross-sectional studies from Ethiopia [36–38, 41–44]. Poor social support was associated with PTSD symptoms, depression, anxiety, and stress [35–38, 42, 43]. Two studies reported that HCWs with perceived stigmatization were more likely to suffer from depression, anxiety, stress, and PTSD [37, 42].

### family concerns

This came up as one of the main risk factors of stress in almost all studies, especially among those HCWs in direct contact with confirmed COVID-19 cases [35–38, 40–45]. A family member suffering from COVID-19 was associated with poor mental health outcomes in HCWs [36, 37].

#### Protective psychosocial factors

Two studies suggest a reduction of perceived stigma can be achieved by sensitization of communities about COVID-19 [37, 42], and four studies recommend solid social support [36, 37, 42, 43].

#### Safety of family

Family safety had the most significant impact in reducing stress. Safety from COVID-19 infection and financial protection of families were essential coping strategies for HCWs [35, 36].

### Underlying illnesses

We found three studies that reported an underlying medical and mental illness as an independent risk factor for poor psychological outcomes [42, 43, 45].

### Protective factors against adverse mental health outcomes

The review identified protective factors to adverse mental health outcomes during COVID-19. The qualitative study from Uganda and four quantitative cross-sectional studies from Ethiopia identified some protective factors [35, 38, 41, 42, 45]. The protective factors are grouped under three thematic areas (i) occupational, (ii) psychosocial, and (iii) environmental aspects.

The qualitative study identified many social coping strategies among respondents, including family networks, community networks, help from family, responsibility to society, assistance from community members, availability of assistance from strangers, and the symbiotic nature of assistance in the community [35].

#### Protective occupational factors

##### Experience

Studies suggest that physicians suffered fewer mental health disorders partly because of their experience with previous epidemics [37, 42, 45].

##### Trainings

Some necessary coping measures include good hospital guidance and ongoing training of frontline HCWs [37, 42, 45].

##### Adequate supply of PPEs

As mentioned above, PPE was a protective factor when adequate and a risk factor for poor mental health outcomes when deemed inadequate [35–37, 42, 43].

## Discussion

The COVID-19 pandemic has been an ongoing global public health emergency that has burdened healthcare workers' physical and mental well-being (HCWs) [1, 5]. Our review confirms the enormous magnitude of mental health impact of COVID-19 on healthcare workers in sub-Saharan Africa, and it is widespread, with significant levels of depression, anxiety, distress, and insomnia; especially those working directly with COVID-19 patients at particular risk [34–37, 39–45]. Out of the twelve articles reviewed, eight studies (66%) came from Ethiopia, and this has implications on the results (Table 1). This finding indicates few research published to date on the psychological impact of the pandemic on the mental health of HCWs in sub-Saharan Africa; a subregion that the COVID-19 pandemic has severely impacted.

### Overview of the study sites

Studies in this review were conducted predominantly in hospital settings. We found only one study relating to primary healthcare workers or facilities [38]. This finding is of concern, as there is increasing evidence that many non-frontline HCWs continue to suffer psychological symptoms long after the conclusion of infectious disease epidemics [7, 8]. In addition, a significant mortality due to COVID-19 was due to excess morbidity, some of which were from primary care facilities. Given that this study is the first narrative review in sub-Saharan Africa, it would be helpful to briefly compare our findings with some published reviews and surveys from other regions (Table 2).

### High prevalence of psychological disorders among participants

Investigators in this review found 16.3–71.9% HCWs with depressive symptoms, 21.9–73.5% had anxiety symptoms, 15.5–63.7% experienced work-related stress symptoms, 12.4–77% experienced sleep disturbances, and 51.6–56.8% PTSD symptoms [35–45]. This high prevalence of mental health symptoms among HCWs in our review is consistent with previous reviews conducted early in the pandemic in sub-Saharan Africa [31], Asia [17, 18, 26, 28], USA & Europe [15, 16], and supported by a batch of cross-sectional studies globally [11–14, 19, 27, 30]. We found mixed results with significant variations within and among regions and countries, as depicted in Tables 1 and 2.

### Risk factors of psychological disorders among participants

Studies established that HCWs responding to the COVID-19 pandemic in sub-Saharan Africa were exposed to long working hours, overworking, exhaustion, high risk of infection, and shortage of personal protective equipment (Tables 3 and 4). In addition, HCWs had deep fear, were anxious and stressed with the high transmission rate of the virus among themselves, high death rates among themselves and their patients, and lived under constant fear of infecting themselves and their families with obvious consequences [35–45]. Some HCWs were deeply worried about the lack of standardized PPEs, known treatments and vaccines to protect against the virus. Many health workers had financial problems, lacked support from families and employers if they contracted the virus [34–37, 39–42, 44]. An additional source of fear and anxiety was the perceived stigma attached to being infected with COVID-19 by the public [36, 41]. Studies found that HCWs, especially those working in emergency, intensive care units, infectious disease wards, pharmacies, and laboratories, were at higher risk of developing adverse mental health impacts compared to others [34–37, 39–44]. This is supported by previous reviews [15–18, 26, 28] and cross-sectional studies [10–14, 20, 21, 23, 25, 30]. However, findings were inconsistent on the impact of COVID-19 on frontline health workers, with ten studies [35–37, 39–42, 44, 45] suggesting they are at higher risk than peers and two studies showing no significant difference in psychological disorders relating to the departments [38, 43].

The Mali’s study was conducted exclusively in primary care facilities among HCWs not involved in treating COVID-19 cases but still registered a very high prevalence of depression 71.9%, anxiety 73.6%, and insomnia 77.0% [39]. In contrast, two studies conducted among HCWs at COVID-19 treatment facilities in Ethiopia [36, 38] registered much lower prevalence of depression 20.2%, anxiety 21.0%, and insomnia12.4% [36], and 16.3%, 30.7% and 15.9% respectively, in the second study [38]. These findings show that not only frontline HCWs experienced mental health disorders during this pandemic but highlight the need for direct interventions for all HCWs regardless of occupation or workstation during this and future pandemics. The significant disparity in the studies could be due to structural, occupational, and environmental issues for example challenges faced by Mali's healthcare systems, characterized by acute equipment shortages, lack of PPEs, human resources, lack of trained and experienced HCWs, ongoing nationwide insecurity, and terrorism compared to Ethiopia. Therefore, local context needs to be considered as contributing factor to mental health disorders among HCWs.

### Regional variations of psychological disorders

Tan et al*.* found a higher prevalence of anxiety among non-medical HCWs in Singapore [27]. As previously noted, the prevalence of poor psychological outcomes varied between countries. Compared to sub-Saharan Africa and China, data from India [23] and Singapore [27] revealed an overall lower prevalence of anxiety and depression than similar cross-sectional data from sub-Saharan Africa [35–45] and China [9, 25, 30]. This finding suggests that different contexts and cultures may reveal different psychological findings and that, it is possible that being at different countries’ outbreak curve may play a part, as there is evidence that it is influential.

Tan et al*.* suggests that medical HCWs in Singapore had experienced a SARS outbreak and thus were well prepared for COVID-19 psychologically and infection control measures [27]. What can be deduced is that context and cultural factors play a role, not just the cadre or role of healthcare workers [16]. It also highlights the importance of reviewing evidence regularly as more data emerge from other countries.

One hospital in Ethiopia found that the thought of resignation was associated with higher chances of mental health disorders and that pharmacists and laboratory technicians who did not receive prior training exhibited higher symptoms of mental health disorders compared to others [36]. Work shift arrangement, considering a dangerous atmosphere presented by working in COVID-19 wards, was one which exacerbated or relieved mental health symptoms among HCWs, with shorter exposure periods being most beneficial [36]. Meanwhile, studies found that financial worries caused by severe lockdowns and erratic payment of salaries and allowances were also major stressors [35]. This finding is like studies in Pakistan [13] and China [30, 32].

In this review, HCWs who had contact with confirmed COVID-19 patients were more affected by depression, anxiety, and stress than their counterparts who had not [35–37, 40, 41, 43, 45]. This finding is like previous reviews [15–18, 26, 28, 31] and cross-sectional studies [9–14, 21, 23–25, 27, 30], which reported higher depression, anxiety, and psychological symptoms of distress in HCWs who were in direct contact with confirmed or suspected COVID-19 patients.

A study in Pakistan showed that 80% of participants expected the provision of PPE from authority [13], and 86% were anxious. Some respondents alluded to forced deployment, while in Mali, 73.3% were anxious, with the majority worrying about the shortage of nurses [39]. Therefore, prospects of being deployed at a workstation where one had not been trained or oriented contributed to fear among health workers. In the sub-Saharan African context, this scenario can best be represented in HCWs involved in internship who must endure hard work during their training. Tan et al*.* found that junior doctors were more stressed than nurses in Singapore [27].

### Socio-demographic characteristics

Nearly all studies in our review suggest that socio-demographic variables for example age, gender, marital status, and living alone or with families contribute to the high mental disorder symptoms [35–37, 39–44]. We, the authors suggest that these observations are handled cautiously as several investigators of these reviewed articles did not entirely control the influence of confounding variables. An alternative explanation for this study's findings may be the more significant risks of frontline exposure amongst women and junior HCWs, predominantly employed in lower-status roles, many of whom lacked experience and appropriate training within healthcare system globally. It is also important to note that respondents to all studies, when disaggregated by gender, and age, were predominantly younger or female, which may have impacted the outcomes of these findings [16]. In addition, the consistently higher mortality rates, and risk of severe COVID-19 disease amongst men would suggest that the complete picture regarding gender and mental health during this pandemic is still incomplete [16]. Moreover, in several studies, both younger and older age groups were equally affected by mental health symptoms but for different reasons. Cai et al*.* [32] in a Chinese study on HCWs for example observed that irrespective of age, colleagues' safety, self and families' safety, the lack of treatment for COVID-19 was a factor that induced stress in HCWs. Similarly, in our review, the lack of PPEs, high infection transmission rates, high death rates among HCWs, and the fear of infecting their families were the factors that induced stress in all HCWs [34–37, 39–45].

We, the authors propose that paying close attention to concerns of HCWs by employers would greatly relieve some stressors and contribute to increased mental well-being of participants. Compared with physicians, our review showed that nurses were more likely to suffer from depression, anxiety, insomnia, PTSD, and stress [35, 37, 39–41, 44, 45]. Workloads and night shifts in healthcare facilities, as well as contacts with risky patients, enhanced nurses' mental distress risks [15–18, 26–28]. In addition, nursing staff have more extended physical contacts and closer interactions with patients than other professionals, providing round-the-clock care required by patients with COVID-19 and thus the increased risk [15]. On the one hand, we posit that most senior physicians are experienced and always keep well-informed with emerging medical emergencies. The majority become aware of emerging epidemic early and actively protect themselves from infections through regular scientific literature updates compared to their junior counterparts. Senior physicians also spend less time in emergency wards unless there is a need to conduct specific procedures which cannot be undertaken by senior housemen or general medical officers. Cai et al*.* [32] concluded that it is essential to have a high level of training and professional experience for healthcare workers engaging in public health emergencies, especially for the new staff. As a result, these findings highlight the importance of focusing on all the frontline HCWs sacrificing to contain the COVID-19 pandemic.

### Regular monitoring of high-risk groups

There is a need to continue monitoring the high-at-risk groups, including nursing staff, interns, support staff, and all deployed in emergency wards. These high-at-risk groups should be encouraged to undertake screening, treatment, and vaccination to avoid the medium and long-term consequences of such epidemics [15, 16, 35, 37, 40, 44].

### Social support and coping mechanisms

The effect of social support and coping measures is in the qualitative study [34] and three other quantitative studies [36, 41, 42] which concluded that respondents with good social support were less likely to suffer from severe depression, anxiety, work-related stress, and PTSD. The qualitative study identified several coping measures, including community and organizational support, family, and community networks, help from family, responsibility to society, and assistance from community members and strangers, including the symbiotic nature of assistance in the community [35]. Other measures include providing accommodation and food to employees [35].

Interestingly, no study examined the association of resilience and self-efficacy with sleep quality, degrees of anxiety, depression, PTSD, and stress. However, a Chinese study by Cai et al. [32] suggests that the social support given to HCWs causes a reduction in anxiety and stress levels and increases their self-efficacy. In divergence, Xiao et al*.* [46] found no relationship between social support and sleep quality.

Only two studies in our review examined the effects of stigma on the mental health of HCWs [36, 41] and found that HCWs with perceived stigma were more likely to be depressed, anxious, stressed, and prone to poor sleep quality [36, 41]. We, the authors suggest that better community sensitization by creating public awareness involving appropriate local community structures and networks are essential. The broader community in sub-Saharan Africa may have suffered severely from infodemics with severe consequences on their mental health, especially during the difficult lockdowns. In addition, removing discrimination/inequalities at the workplace based on race and other social standings have a powerful influence on the mental health outcomes of HCWs. Also, because emotional exhaustion is long associated with depression, anxiety, and sleep disturbances, none of the studies in our review examined burnout as an essential component of mental health disorders in HCWs in sub-Saharan Africa.

### Protective and coping measures

In this review we have provided evidence about personal, occupational, and environmental factors that were important protective and coping measures against psychological disorders. Based on these factors we suggest some protective and coping measures which can help to reduce the negative effects of the pandemic on mental health of HCWs in sub-Saharan Africa. Organizations and healthcare managers need to be aware that primary prevention is key to any successful interventions to contain and control any epidemic. This should take the form of planned regular training, orientation and continuing medical education grounded on proven infection control measures. These measures need to be backed up by timely provision of protective equipment, drugs, testing facilities, vaccines, isolation facilities, clinical and mental health support, and personal welfare of HCWs [35–37, 42, 45]. The effect of community and organizational support and coping measures was shown by the qualitative study [35] and five other quantitative studies [36, 37, 41–43] indicating that respondents who had good social and organizational support were less likely to suffer from severe depression, anxiety, work related stress and PTSD. Prior experience with comparable pandemics and training are suggested as beneficial coping strategies for healthcare workers during this pandemic but also local social structural and geopolitical conditions appear to determine the pattern and evolution of mental health symptoms among HCWs [14, 15, 31, 32, 47]. In our case the high prevalence of all mental health symptoms in non-frontline primary health care facilities in Mali [39] which was already plagued with instability and weak healthcare systems prior to the pandemic is a case in point. Results are particularly consistent in showing that provision of PPEs, testing kits, orientation training of workers, work shift arrangements, provision of online counselling, provision of food and accommodation and prompt payment of allowances by employers were important protective measures [35–39, 41–47]. The feeling of being protected is associated with higher work motivation with implication for staff turnover [35, 38, 43, 45]. Hence, physical protective materials [14], together with frequent provision of information, should be the cornerstone of any interventions to prevent deterioration in mental health of HCWs (Table 5). Finally, provision of rest rooms, online consultation with psychologists/psychiatrists, protection from financial hardships, access to social amenities and religious activities are some important coping measures [35, 36, 38, 42, 45]. In this era of digital health care with plentiful internet and smartphones, organization can conduct online trainings, online mental health education, online psychological counselling services, and online psychological self-help intervention tailored to the needs of their HCWs [35, 37, 42]. In addition, it is essential to understand and address the sources of anxiety among healthcare professionals during this COVID-19 pandemic, as this has been one of the most experienced mental health symptoms [48]. Adequate protective equipment provided by health facilities is one of the most important motivational factors for encouraging continuation of work in future outbreaks. Furthermore, availability of strict infection control guidelines, specialized equipment, recognition of their efforts by facility management, government, and reduction in reported cases of COVID-19 provide psychological benefits [15, 32]. Finally, we call upon Governments (the largest employers of HCWs) in sub-Saharan Africa to do what it takes to improve investments in the mental health of HCWs and plan proactively in anticipation of managing infectious disease epidemics, including other expected and unexpected disasters.

### Future research direction

There was no study that examined the association of resilience and self-efficacy with sleep quality, degrees of anxiety, depression, PTSD, and stress. Although emotional exhaustion has long been associated with depression, anxiety, and sleep disturbances, no study in our review examined burnout as an important component of mental health disorders in HCWs in sub-Saharan Africa. The impacts of infodemics, stringent lockdown measures, discrimination/inequalities at workplaces based on race, and other social standings on mental health outcomes of HCWs need to be investigated.

Future studies are needed on the above including other critical areas like suicidality, suicidal ideations, and substance abuse during the COVID-19 pandemic. In addition, there is a significant variation of related literature calling for more rigorous research in future. More systematic studies will be required to clarify the full impact of the pandemic so that meaningful interventions can be planned and executed at institutional and national levels in the Sub-Saharan Africa.

### Limitations of this study

There are some limitations to this study. First, most of the studies are from one country, limiting the generalizability of the results to the whole African continent. Second, all the studies were cross-sectional and only looked at associations and correlations. There is a need for prospective or retrospective cohort or case–control studies on this subject matter. Longitudinal research studies on the prevalence of mental disorders in the COVID-19 pandemic in the sub-Saharan Africa are urgently required. Third, most studies reviewed did not adequately examine protective factors or coping measures of the health workers in their settings. In addition, most studies did not pay strict attention to confounding variables which could have led to inappropriate results and conclusions. Fourth, most sample sizes were small and unlikely representative of the population and yet larger sample sizes would better identify the extent of mental health problems among health workers in the region. Fifth, depression, anxiety, and stress were assessed solely through self-administered questionnaires rather than face-to-face psychiatric interviews. Sixth, these studies employed various instruments and different cut-off thresholds to assess severity. Notably, the magnitude and severity of reported mental health outcomes may vary based on the validity and sensitivity of the measurement tools. Seventh, there was no mention of mental baseline information among the studied population and therefore it was unknown if the studied population had pre-existing mental health illnesses that decompensated during the pandemic crisis. Eight, investigators did not give much attention to stigma, burnout, resilience, and self-efficacy among study participants.

Furthermore, our review did not employ systematic reviews or meta-analyses methods for the information generated. This narrative review paper precluded deeper insight into the quality of reviewed articles for this paper. Still, our observation was that investigators did not consider the strict lockdown measures, quarantine, and isolation imposed by many countries in sub-Saharan Africa as possible risk factors for mental health disorders among HCWs.

## Conclusion

Based on the articles reviewed, the prevalence of depression, anxiety, insomnia, and PTSD in HCWs in the sub-Saharan Africa during the COVID-19 pandemic is high. We implore health authorities to consider setting up permanent multidisciplinary mental health teams at regional and national levels to deal with mental health issues and provide psychological support to patients and HCWs, always supported with sufficient budgetary allocations.

Long-term surveillance is essential to keep track of insidiously rising mental health crises among community members. There is a significant variation of related literature thus calling for more rigorous research in the future. More systematic studies will be needed to clarify the full impact of the pandemic so that meaningful interventions can be planned better and executed at institutional and national levels in sub-Saharan Africa.

## Data Availability

Datasets analysed in the current study are available from the corresponding author at a reasonable request.

## Abbreviations

COVID-19: Coronavirus disease 2019
HCWs: Healthcare workers.
MH: Mental health
PHE: Public health emergency
PPE: Personal protective equipment
WHO: World Health Organisation
